# Botanicals as modulators of depression and mechanisms involved

**DOI:** 10.1186/s13020-019-0246-9

**Published:** 2019-07-15

**Authors:** Zhengrong Zhang, Taomei Deng, Manli Wu, Aisong Zhu, Guoqi Zhu

**Affiliations:** 1 Key Laboratory of Xin’an Medicine, Ministry of Education, Anhui University of Chinese Medicine, Meishan Road 103, Hefei, 230038 China; 2 School of Pharmacy, Anhui University of Chinese Medicine, Hefei, 230038 China; 3 College of Basic Medicine, Zhejiang Chinese Medical University, Hangzhou, 310053 China

**Keywords:** Depression, Botanicals, Mechanisms, HPA, Inflammation

## Abstract

Depression is the most disastrous mood disorder affecting the health of individuals. Conventional treatments with chemical compounds for depression have limitations, while herbal medicine has unique therapeutic effects. This paper introduces the pharmacological basis and biological mechanisms underlying the botanical antidepressants over the past 5 years. Based upon the specific therapeutic targets or mechanisms, we analyzed the pathological roles of monoamine neurotransmitters, the hypothalamic–pituitary–adrenal axis, inflammation, oxidative stress, synaptic plasticity performed in antidepressant of the botanicals. In addition, gut flora and neurogenesis were also preferentially discussed as treatment approaches. Based on the complex pathogenesis of depression, we suggested that mixed use of botanicals, namely prescription would be more suitable for treatment of depression. In addition, neural circuit affected by botanicals or active components should also attract attention as the botanicals have potential to be developed into fast-acting antidepressants. Finally, gut flora might be a new systemic target for the treatment of depression by botanicals. This review would strength botanical medicine as the antidepressant and also provides an overview of the potential mechanisms involved.

## Background

**Fig. 1:**
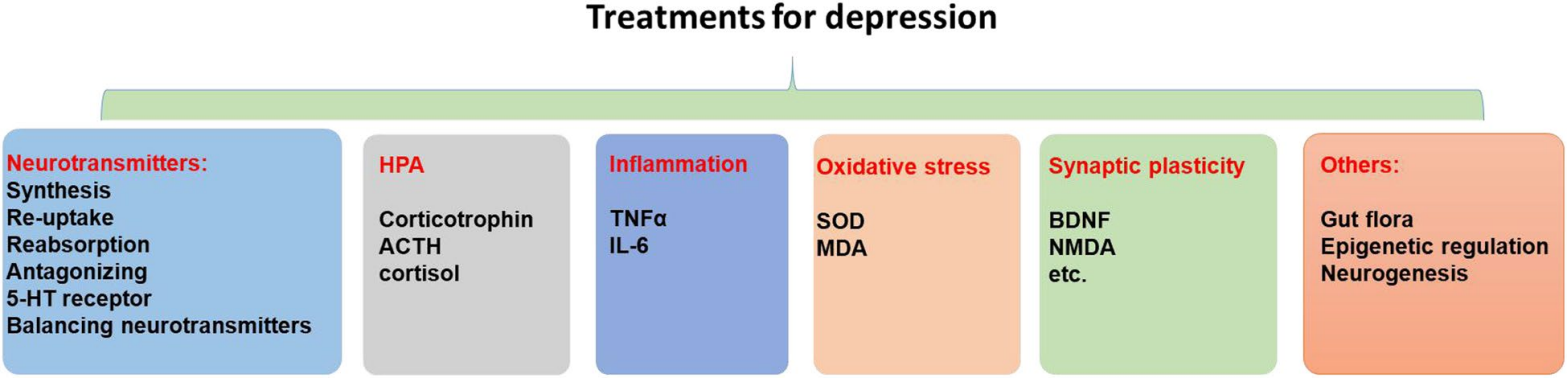
Potential treatment targets for depression based on the pathogenesis. Treatments targets for depression include neurotransmitters, HPA, inflammation, oxidative stress, synaptic plasticity and others (gut flora, epigenetic regulation and neurogenesis)

Depression is a devastating psychiatric disorder, generally characterized by loss of interest, anxiety, sleep disturbance, lack of energy, and suicidal thoughts. Epidemiological studies show that the global prevalence of depression and depression-related symptoms is increasing annually [1]. The prevalence of depression is high in women (20% to 25%), while is relatively low in men (7% to 12%) [2]. Depression is one of the major causes for suicide. However, the cause of depression is unclear, and the factors causing the disease are complicated. Current pathogenesis includes abnormal expression of neurotransmitters [serotonin (5-HT), norepinephrine (NE) and dopamine (DA)] or receptors, the hypothalamic–pituitary–adrenal (HPA) axis dysfunction, imbalance of inflammatory cytokines, oxidative stress, impairment of synaptic plasticity [3, 4]. In addition, abnormality of gut flora and epigenetic alteration of genes are also important determinants for the symptoms of depression [5, 6] (Fig. 1). However, the drugs available for depression were restricted in regulating neurotransmitters, including selective serotonin reuptake inhibitors, serotonin-norepinephrine reuptake inhibitors, atypical antidepressants, tricyclic antidepressants and monoamine oxidase inhibitors.

Animal models were produced mainly based on the symptoms of depression, including cognition and emotion, behavioral despair, hopelessness, anxiety-like symptoms, anxiety and locomotor activity, and anhedonia [7]. Learned helpless (LH) model, unpredictable chronic mild stress (UCMS) model, early life stress model, olfactory bulbectomy (OBX) model, social defeat model, chronic restraint stress (CRS) model and glucocorticoid/corticosterone model are typical models which are widely used to investigate the pathogenesis and screen therapeutic agents for depression [7]. Additionally, several genetic depression models were also utilized (Tph1^−/−^ mice, Vmat2^−/−^ mice, etc.) [8, 9].

Herbal products are the major constitute of traditional Chinese medicine, which embodies intact theory to treat diseases [10]. Botanicals or their active components have been extensively investigated in the treatment of depression-like behaviors [11]. Especially, the mixed use of botanicals, namely prescription in Chinese medicine has a prior function to ameliorate symptoms of depression [12]. With the discovery of the pathogenesis of depression, therapeutic targets for botanicals have been gradually verified using the depressive animal models. This review comprises of a systematic 5-year update of research of botanicals for the treatment of depression based on the pathogenesis and potential therapeutic targets for depression.

## Neurotransmitters and their receptors

Depression has been chemically linked to problems or imbalances in the brain with regard to the neurotransmitters like 5-HT, NE, and DA [13]. Remedy of the depressed neurotransmitters has thus become the primary selection for treatment of depression. The active components from botanicals have the advantages to remedy the abnormalities of neurotransmitters through regulating synthesis of neurotransmitters, reabsorption of neurotransmitters, balancing the ratio of excitatory and inhibitory neurotransmitters, re-uptake of neurotransmitters by neurons, antagonizing 5-HT2A receptor, etc.

Tian et al. [14] found that adhyperforin, a newly-identified active component of *H. perforatum* exerts strong antidepressant effects by binding to 5-HT and NE transporter and inhibiting their reabsorption. Zirak et al. [15] showed that the anti-depressant effects of hypericin may be related to reduction of NE and 5-HT in the brain. Further, Ji et al. [16, 17] demonstrated that the essential oil from *P. frutescens* (EOPF) relieved depression-like behaviors in UCMS rats, likely through reversing changes in 5-HT and 5-hydroxyindoleacetic acid (5-HIAA) concentrations. The antidepressant effect of saffron is attributed to the activities of safranal and crocin through the re-uptake of DA, 5-HT, and NE from neurons [18]. In experiments to assess the effects of safranal and crocin on levels of catecholamine and 5-HT in the brain, crocin was demonstrated to be a non-competitive inhibitor of monoamine oxidase (MAO)-A and MAO-B, while safranal did not act on these two isomers [19]. Extract of *C. tubulosa* can modulate the concentrations of acetate, as well as hexanoic acid, to restore levels of 5-HT in UCMS rats. Oh et al. found that leaf extract from *V. bracteatum* exerted antidepressant-like effects through regulation of monoaminergic systems and glucocorticoids with neuroprotective effects, alongside antagonism of the 5-HT2A receptor. Furthermore, *V. bracteatum* exerts neuroprotective effects by decreasing protein levels of MAO-A and serotonin transporter (SERT), and increasing those of tryptophan hydroxylase 2 (TPH2), through upregulation of the extracellular-regulated kinase (ERK)/Akt signaling pathway [20]. Ginsenoside Rb1 and its metabolite, compound K, ameliorate depression-like behaviors in female mice by regulating the 5-HT2A-receptor [21, 22]. *Rhodiola* has beneficial effects on learning and memory in neonatal rats, through modulation of acetylcholine levels and MAO inhibitory activity [23, 24]. Curcumin has antidepressant effects, which may be related to the inhibition of MAO and enhancement of monoamine neurotransmitters [25]. The antidepressant effects of silymarin may also be due to the decrease of monoamine synthesis (5-HT, NE, etc.) in the hippocampus and cerebral cortex of mice with UCMS-induced depression-like behavior [26, 27].

**Fig. 2:**
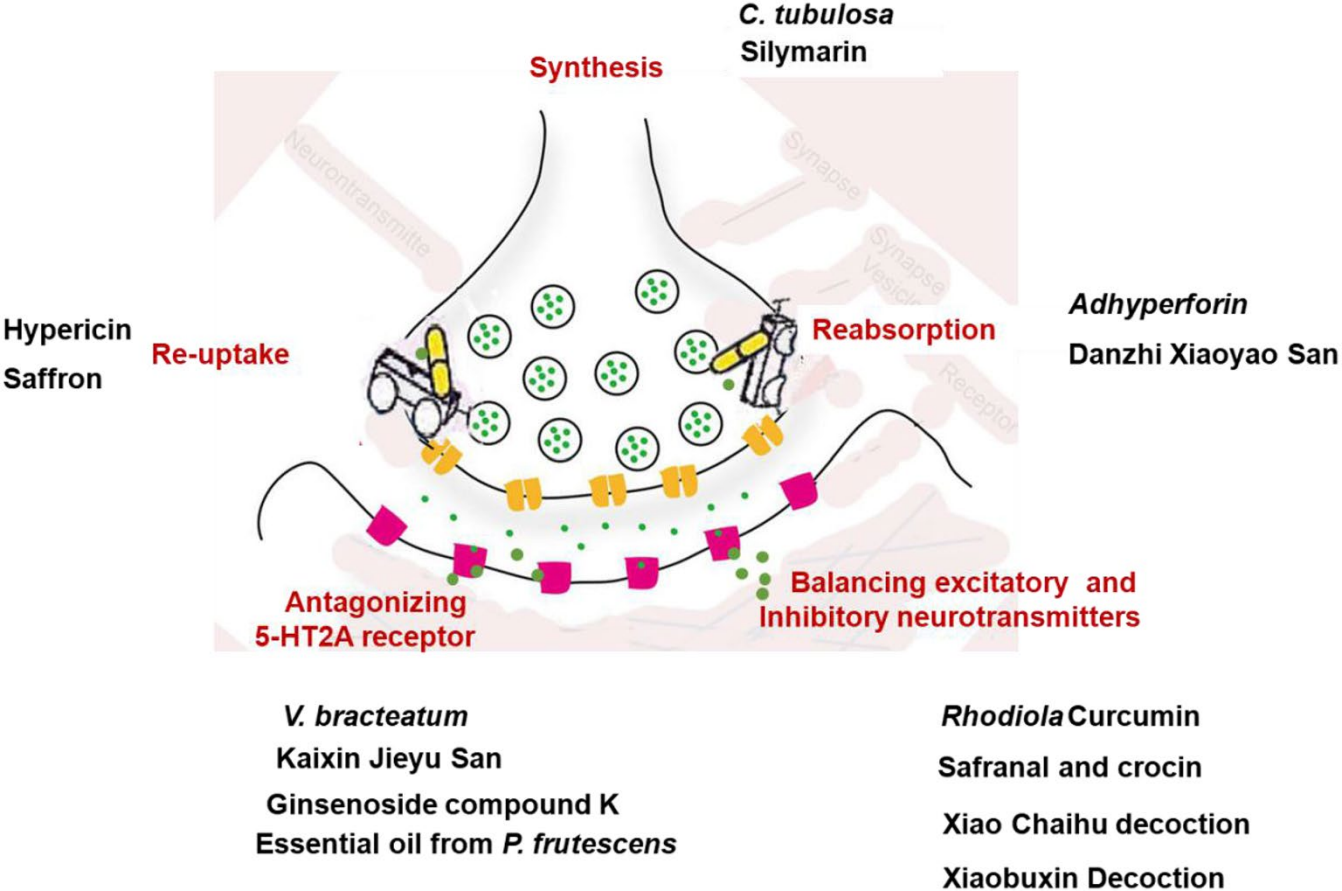
Botanicals and active components for the treatment of depression with the aspect of neurotransmitters. *C. tubulosa* and silymarin influence synthesis of neurotransmitters; hypericin and saffron affect re-uptake of neurotransmitters by neurons; adhyperforin and Danzhi Xiaoyao San effect on reabsorption of neurotransmitters; *V. bracteatum*, Kanxin Jieyu San, etc. could antagonize 5-HT receptor; Rhodiola curcumin etc. could balance excitatory and inhibitory neurotransmitters

In addition to the active components, Chinese formula revealed prior activities in regulating neurotransmitters. Ma et al. found that Xiao Chaihu decoction exerts antidepressant effects by increasing the level of monoamine neurotransmitters in mouse hippocampus microdialysis solution, in mice subjected to social isolation, and inhibiting the conversion of 5-HT (5-HIAA/5-HT) [28]. In depressive mice, the expression of monoamine neurotransmitter synthase (TPH2 and TH) is enhanced, while that of SERT is inhibited, and the expression of hippocampal monoamine neurotransmitter synthase reduced [29]. Yang et al. [30] also demonstrated that Chaihu Shugan San can effectively improve the symptoms of depression by increasing 5-HT1A receptor expression in the dentate gyrus of the hippocampus in epileptic rats with depression. Huang et al. [31] focused on the expression of monoamine neurotransmitters and 5-HT receptor subtypes and found that Kaixin Jieyu San could normalize 5-HT and NE levels and regulate the balance of 5-HT1A and 5-HT2A receptor expression in rat brain. Wu et al. [32] showed that Danzhi Xiaoyao San can ameliorate depression-like behaviors in a UCMS-induced rat model. The mechanism underlying the effects of Danzhi Xiaoyao San against depression involves regulation of monoamine levels and amino acid neurotransmitters in the hippocampus. Zhang et al. [33] showed that the antidepressant action of flavonoids in Xiaobuxin Decoction is related to the regulation of extracellular serotonin levels the in central nervous system, and inactivation of the rate of limiting enzyme in the synthesis of 5-HT and tryptophan hydroxylase (Fig. 2).

## Hypothalamic–pituitary–adrenal axis

HPA axis is an interactive neuroendocrine unit comprising of the hypothalamus, the pituitary gland, and the adrenal glands. The HPA axis has been revealed in pathophysiology of a series of mood and cognitive disorders [34]. Hyperactivation of HPA axis is thought to be a major cause of major depression [35]. Botanicals and their active components have been extensively investigated regarding their functions in regulating HPA axis in depression. EGb761, catalpol, geniposide, *R. glutinosa,* Xiao Chaihu decoction, Danzhi Xiaoyao San have been reported to normalize the HPA axis in depression [32, 36–39]. The anti-depressive effects of ginsenoside Rg1 are mainly through improvement of corticosterone and testosterone levels, modulating protein levels of glucocorticoid receptor (GR) and androgen receptor (AR), and mediating recovery of the HPA axis [40]. Moreover, geniposide can also upregulate GRα expression in the hypothalamic paraventricular nucleus to treat depression-like behaviors [41]. Therefore, GR in hypothalamic paraventricular nucleus is potential target for repair of HPA in depression. Saikosaponin A can also have antidepressant-like effects, by inhibiting hyperactivity of the HPA axis [42]. It can also possible that botanicals and their active components prohibit inflammation, which subsequently eliminates HPA axis hyperactivation [43]. In depression, HPA axis is out of control due to a down-regulation of its negative feedback controls. Corticotrophin is hypersecreted from the hypothalamus and triggers the release of adrenocorticotropic hormone (ACTH) from the pituitary and uncontrollable release of cortisol [44]. Thereafter, cortisol receptors become desensitized leading to increased activity of the pro-inflammatory immune mediators and disturbances in neurotransmitter transmission [45]. The impairment of HPA could also damage neuronal synaptic transmission or neurogenesis, which contributes to depression-like behaviors. For an example, Li et al. [46] reported that Saikosaponin D can counter UCMS-induced depressive behaviors in rats by increasing the phosphorylation of cAMP response element-binding protein (CREB) and promoting brain-derived neurotrophic factor (BDNF) expression, which was mediated by enhancement of HPA axis function and consolidation of hippocampal neurogenesis. Nevertheless, the exact mechanisms underlying the recovery of the HPA axis by botanicals still require clarification.

## Inflammation

Evidence is accumulating to show that depression and inflammation are closely connected and may fuel each other [45]. Anti-inflammation has become an important stratagem for treatment of depression. Varieties of botanicals have the potential to anti-inflammation and ameliorate the depression-like behaviors. Astragaloside IV (ASIV), ginsenosides, quercetin, naringenin, saikosaponin A, EGb761, resveratrol, *T. lythroides*, curcumin, Rhizoma Gastrodiae, Xiaobuxin decoction were well-known for their anti-inflammation in depressive models [21, 22, 36, 47–57].

The antidepressant effects of ASIV are also associated with modulation of neuroinflammation via promotion of peroxisome proliferators-activated receptors γ expression [58]. Quercetin also suppresses oxidative-nitrosamine stress mediated neuroinflammation, via tumor necrosis factor-α (TNF-α) and interleukin 6 (IL-6), and showed neuroprotective effects through the microglial inhibitory pathway [59]. Zhang et al. reported that EGb761 can attenuate depression-like behaviors induced by long-term light avoidance treatment in mice. The underlying mechanism may be associated with inactivation of nuclear factor-κB (NF-κB) signaling pathway-related inflammation in the hippocampus [60]. In a rodent model of CRS-induced depression, *P. Ginseng* upregulated the Nrf2-heme oxygenase-1 pathway and down-regulates the neuroinflammatory system (MAPK and NF-κB pathways) in the amygdala [61]. Li et al. [62] suggested that Xiaoyao San can alleviate hippocampal neuronal injury and reverse effects measured using the hypertension labyrinth test, through activation of the TNF-α/Janus Kinase 2/Signal Transducer and Activator of Transcription 3 (JAK2/STAT3) pathway in a rat model of chronic immobilization stress-induced anxiety.

## Oxidative stress

Causative factors for major depression include inflammation, autoimmune tissue damage and prolonged psychological stress, which lead to oxidative stress [63]. Inflammation and damage of mitochondria generate free radicals. With the accumulation of free radicals or consume of antioxidant system, reactive oxygen species (ROS) react with macromolecules (fatty acid, DNA, protein, etc.) and cause damage to these macromolecules. Brain is one of the most vulnerable organs to the damaging effects of ROS, which may explain ROS involvement in several neuropsychiatric diseases, especially depression [64]. To this end, anti-oxidative stress is also supposed as a treatment stratagem for botanicals. Zhao et al. [36] found that EGb761 can ameliorate lipopolysaccharides (LPS)-induced depression-like behaviors possibly through reduction of oxidative stress. With a strong anti-oxidative ability, ginsenoside Rg3 [65, 66], ASIV [47–49], geniposide [67], saikosaponin [46], resveratrol [68], quercetin [50, 51], naringenin [52], *Thymelaea lythroides* [53], *Polygala japonica* [69], Rhizoma Gastrodiae [56, 70], silymarin [71–74] may also ameliorate depression-like behaviors through the anti-oxidative action.

**Fig. 3:**
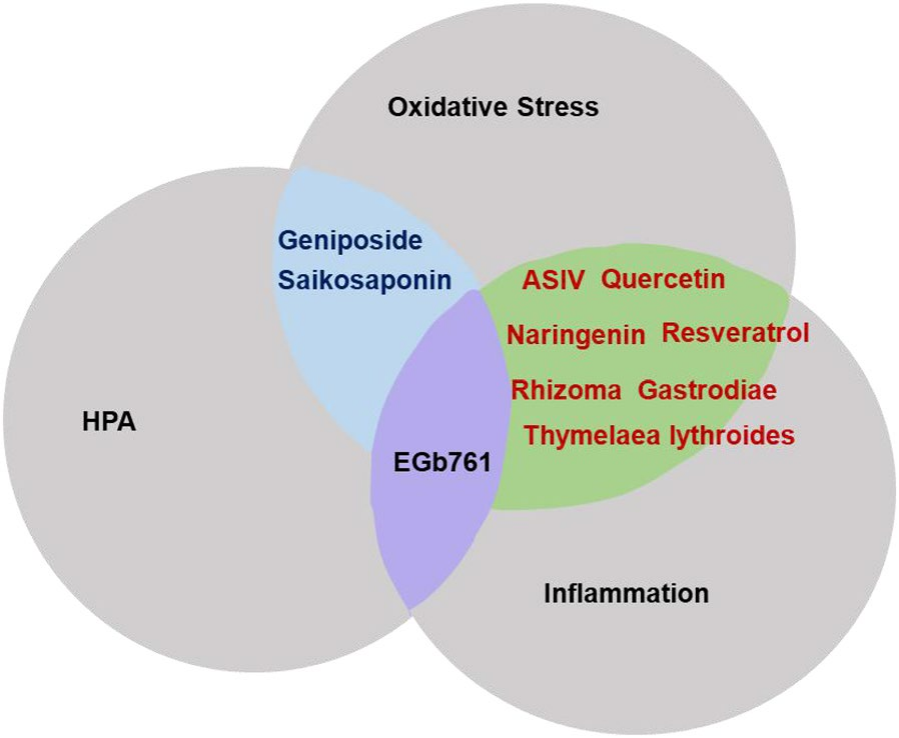
Interplay of oxidative stress, HPA and inflammation involved in the effects of botanicals and active components on depression

Oxidative stress plays a crucial role in the development of inflammation and anti-oxidants thus could prohibit inflammation. Vice versa, inflammation could also initiate oxidative stress [63]. The interrelationship between inflammation and oxidative stress explain that most botanicals exert anti-depressive action through inhibiting both inflammation and oxidative stress [50, 51, 55, 75] (Fig. 3).

## Synaptic plasticity

Synaptic plasticity is one of the most important physiological features of neurons [76]. It is not only related to memory, motor, etc., but also the important determinant of psychiatric disorders. In fact, synaptic regulation has been proposed as one of the most important mechanisms to find antidepressants [77]. Synaptic regulators, such as BDNF/tropomyosin receptor kinase B (TrkB), *N*-methyl-d-aspartate (NMDA), glutamate, estrogen, insulin, or their downstream signaling pathways, like PI3K/AKT/mTor are crucial therapeutic targets for depression [78]. In recent years, botanicals have attracted extensive attention regarding their functions in synaptic plasticity in depression models. Therefore, synaptic plasticity has been proposed as new insights for screening antidepressants, especially rapid-acting antidepressants [79, 80].

### BDNF

**Fig. 4:**
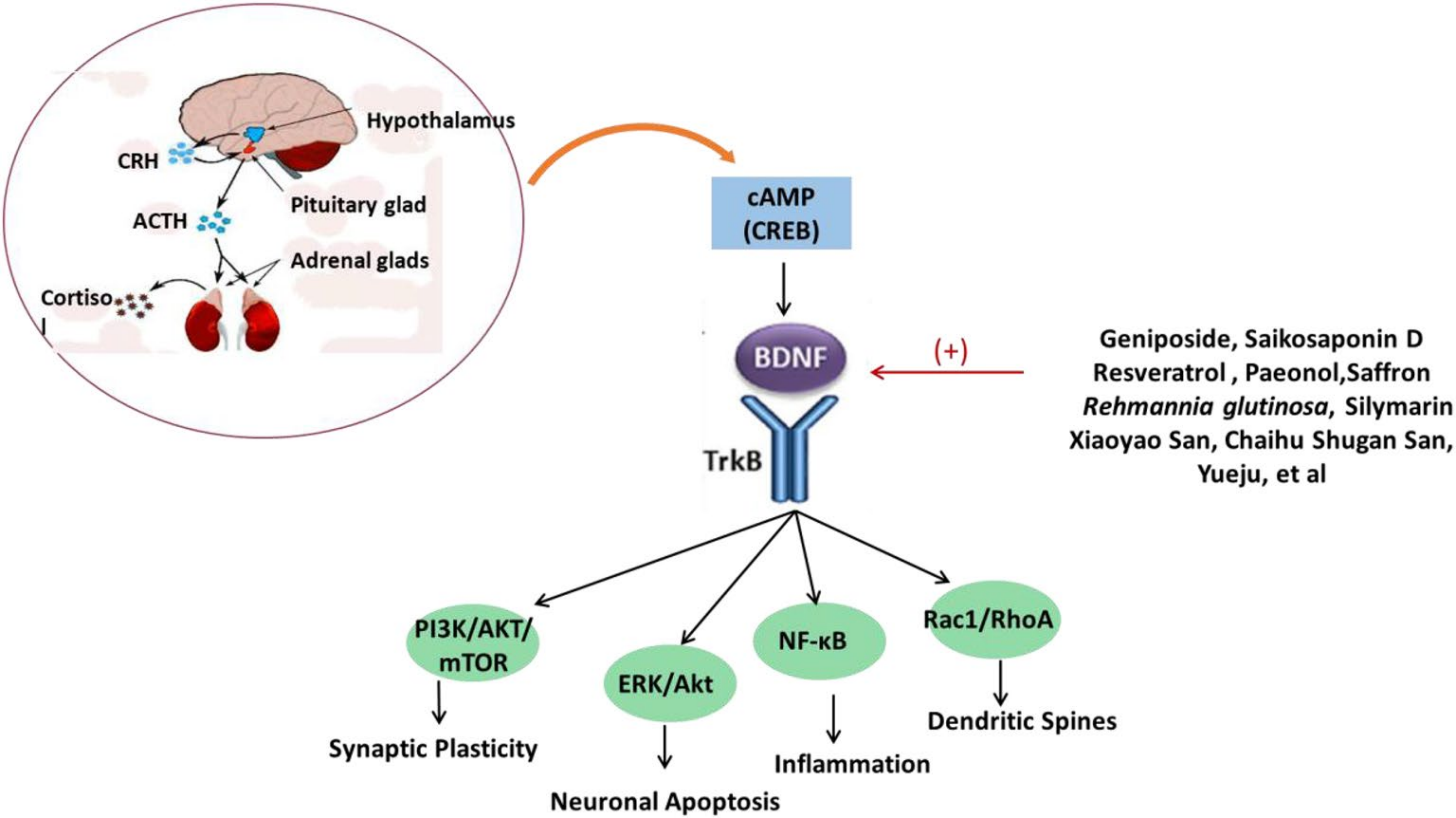
The synthesis of BDNF and potential downstream involved in the effects of botanicals and active components on depression. In brain, botanicals and active components promote BDNF synthesis through cAMP-PKA-CREB signaling pathway. The downstream of BDNF/TrkB pathway include PI3 K/AKT/mTOR-regulated synaptic plasticity, ERK/AKT-regulated neuronal apoptosis, NF-κB-regulated inflammation and Rac1/RhoA-regulated dendritic spine genesis

Geniposide [67], saikosaponin D [46], resveratrol [81, 82], paeonol [83, 84], ginsenosides [85–87], geniposide [67], naringenin [88], *Perilla* seed oil [89], the water extract of saffron [90], catalpol [37], extract of *C. tubulosa* [91], *Rehmannia glutinosa* [38], silymarin [92], Xiaoyao San [93], Chaihu Shugan San [94], Yueju [95], etc. could prevent depression-like behaviors through increasing BDNF expression. At present, our lab also found that curculigoside prevented depression-like activities through increasing hippocampal BDNF level [96]. Interestingly, most of botanicals and active components facilitate BDNF expression through promoting cAMP/PKA/CREB signaling way. Yu et al. revealed that ginsenoside Rg1 has neuroprotective and antidepressant roles through activation of the CREB/BDNF system in the basolateral amygdala and regulation of the synapse-associated factor, miR-134, in a rat model of depression [97]. Botanicals and active components increase BDNF expression, thereafter activating BDNF/TrkB-ERK/Akt to regulate neuronal apoptosis [94], BDNF-Rac1-RhoA pathway to regulate genesis of dendritic spines [83], and BDNF/TrkB/NF-κB pathway to regulate inflammation [84] (Fig. 4).

### NMDA

Accumulating evidence indicates that NMDA receptors are involved in the pathophysiology of depression and implicated as therapeutic targets [98]. In an olfactory bulbectomy model, the antidepressant effects of quercetin act through reinforcement of NMDA receptor inhibition, synthesis of nitric oxide, and reduction of lipid hydroperoxide content in the hippocampus [99]. Xia et al. found that Yueju may confer acute and long-lasting antidepressant effects by favorably modulating the function of NMDA receptors in the hippocampus however, its antidepressant effects were different from those of ketamine, in that Yueju was not influenced by blockade of amino-3-hydroxy-5-methyl-4-isoxazole propionate receptor [100].

## Others

### Gut flora

Gut flora is the complex community of microorganisms that live in the digestive tracts of humans and animals. Gut flora was not only related to food digestion and gastrointestinal diseases, but also modulates a variety of diseases, including psychiatric disorders [101]. Recent advances point that botanicals and active components regulate gut flora to ameliorate depression-like behaviors, including Xiaoyaosan [102], berberine [103], resveratrol [104], *Cistanche tubulosa* extract [105]. The gut metabolites, including l-threonine, isoleucine, alanine, serine, tyrosine, and oxidized proline were supposed as the major cause for depression-like behaviors [106, 107]. Gut–brain-axis was also thought to one of the mechanisms for depression [108, 109].

### Neurogenesis

Neurogenesis is important way for the recovery of neurodegenerative diseases, including Alzheimer’s disease, Parkinson’s disease, and stroke [110]. Nevertheless, neurogenesis was also reported as a useful method to ameliorate depression by botanicals. Saikosaponin D can counter UCMS-induced depressive behaviors in rats by promoting hippocampal neurogenesis [46]. The aqueous extract of *P. japonica* can alleviate depression-like behaviors by stimulating neurogenesis in the adult dentate gyrus. Silymarin may also promote neurogenesis in the hippocampus and cerebral cortex of mice with UCMS-induced depression-like behavior [26, 27]. Xiao Chaihu decoction may also promote neurogenesis in CORT-induced depression mouse model [39]. Gao et al. [111] demonstrated that Xiaoyao San reduced depression-like behaviors in a CUMS-induced depression model by improving hippocampal neurogenesis and reversing cerebral blood oxygen level-dependent (BOLD) activation. Pan et al. [112] showed that Kaixin Jieyu San functioned to reduce depressive behavior and improve cerebral hypoperfusion, which may be related to up-regulation of neurogenesis and balance of the fibrinolysis system.

## Future prospects

This paper summarizes the therapeutic effects of botanicals on depression, with the aim of providing information about drugs for use in clinical practice. We also concluded and detailed the potential therapeutic targets for botanicals. According to the literatures, botanicals and their active components could fight against depression from the following aspects: neurotransmitters and receptor, inflammation, HPA axis, oxidative stress, synaptic plasticity, and others. These information provide that botanicals have broad therapeutic targets for depression, implicating valuable significance to develop anti-depressants from botanicals.

Medication for depression includes selective serotonin reuptake inhibitors, serotonin-norepinephrine reuptake inhibitors, atypical antidepressants, tricyclic antidepressants and monoamine oxidase inhibitors. However, antidepressant medications also come with strong side effects and safety concerns, and withdrawal can be very difficult. Interestingly, some botanicals and active components have the effects to regulate neurotransmitters. As we all know, active components or botanicals are naturally- formed. Toxicity or side effects are relatively mild. Therefore, the active components possess the potential to be developed into antidepressants. Moreover, besides regulating neurotransmitters, some botanicals and active components (like curcumin) also have other pharmacological activities, such as antioxidative, anti-inflammation, and regulating synaptic plasticity. These agents would be more suitable to be developed into antidepressants because of the complex pathogenesis of depression [113]. Interestingly, some prescriptions exhibit superior antidepressive activity through regulating multiple pathways or cascades. For an example, Xiao Chaihu decoction is described in the book, “*Treatise on Febrile and Miscellaneous Diseases*”, by Zhang Zhongjing. It is composed of *Bupleurum chinense*, Radix Scutellariae, Ginseng, *Pinellia ternata*, *Glycyrrhiza uralensis*, Ginger, and Jujube. The components of the prescription could balance neurotransmitters, ameliorate HPA axis, regulate synaptic plasticity to treat depression [28, 29, 39]. Moreover, some active components from the prescription could also fight against oxidative stress. Therefore, Xiao Chaihu decoction, Xiaoyao San, Chaihu Shugan San, Kaixin Jieyu San, Danzhi Xiaoyao San, Xiaobuxin decoction should attract more attentions to treat depression [28, 32, 33, 62, 112, 114–117]. On the one hand, the complex pathogenesis of depression would benefit from the multiple components with the corresponding targets. On the other hand, depression is featured by different complications. The prescription of different botanicals would better treat depression based upon the theory of syndrome differentiation and treatment [28].

At present, most of the studies reported BDNF/TrkB signaling pathway as the therapeutic target for depression by the botanicals, which points out the importance of this specific signaling pathway in the pathogenesis of depression [118]. BDNF/TrkB is a crucial synaptic regulator, which not only correlates with memory but also with mood disorders [96, 119]. Therefore, this target should be kept to screen antidepressants. BDNF is a good factor which could nutrition neurons, however with a chronic effect. Depressive-like behaviors, especially depression-related suicide happen fast. Acute antidepressants like ketamine are also urgent or of more significance to fight against depression-related suicide [120]. With the development of photogenetic technology, optical fiber recording technology, neural circuits involved in depression-like behaviors have gradually been discovered [121]. Therefore, we are required to continue seeking the botanicals from the traditional regulators of synaptic. Moreover, these novel techniques should also be applied to screen the potential active components from botanicals which could influence neural circuit involved in depression [122, 123].

## Conclusion

As we described above, gut flora is an advanced and hot mechanisms for treatments of brain diseases. From the aspect of gut flora, the holistic view of Chinese medicine could be better reflected. In addition, other changes including epigenetic modification should also be paid more attention, as depression was also supposed as a systemic disease, which is not only related to brain. In the future, with the application of genome-wide investigation techniques, genomics technology, and systems biology, it will be helpful to identify new targets and mechanisms for treatment of depression by verifying different pathways and targets and revealing the biological basis of this condition.

## Data Availability

All reported or analyzed data in this review is extracted from published articles.

## Abbreviations

5-HT: 5-hydroxytryptamine
5-HIAA: 5-hydroxyindoleacetic acid
ACTH: adrenocorticotropic hormone
AR: androgen receptor
ASIV: astragaloside IV
BOLD: blood oxygen level-dependent
BDNF: brain-derived neurotrophic factor
CREB: cAMP responsive element-binding protein
CRS: chronic restraint stress
DA: dopamine
EOPF: essential oil from *P. frutescens*
ERK: extracellular-regulated kinase
GR: glucocorticoid receptor
HPA: hypothalamic–pituitary–adrenal
IL-6: interleukin 6
JAK2/STAT3: Janus Kinase 2/Signal Transducer and Activator of Transcription 3
LH: learned helpless
LPS: lipopolysaccharide
MAO: monoamine oxidase
MDA: 3,4-methylene dioxy amphetamine
NF-κB: nuclear factor-κB
NMDA: *N*-methyl-d-aspartic acid
OBX: olfactory bulbectomy
ROS: reactive oxygen species
NE: norepinephrine
SERT: serotonin transporter
SOD: superoxidase dismutase
TPH2: tryptophan hydroxylase 2
TNF-α: tumor necrosis factor-α
TrkB: tropomyosin receptor kinase B
UCMS: unpredictable chronic mild stress
